# XDR-TB in South Africa: Back to TB Sanatoria Perhaps?

**DOI:** 10.1371/journal.pmed.0040160

**Published:** 2007-04-24

**Authors:** Ramalitse Sakoane

It is only about 25 years since the abolition or abandonment of TB sanatoria in South Africa. It is likely that there are people in South Africa who are familiar with the idea of isolation of TB patients from the general public emanating from the era of TB sanatoria, and it is conceivable that they are likely to be understanding of the reasons that were used to keep patients there during their treatment.

These people could help to make the idea of isolation in sanatoria acceptable again to the general public. Anyway, the TB sanatoria of this era were rightfully regarded as hospitals, as indeed they were, and I cannot see why modern day sanatoria should be viewed any differently. Granted, with the likelihood that the patients detained in these envisaged isolation areas may be more likely to die than be discharged, it will be a mammoth task to convince the “detainees” to stay there or their families to allow them to be kept there, especially if they are minors or the elderly infirm. It is a tough call but it is worth a try.

The South African government owes it to the South African public to explore this idea or one along these lines. There is no time for chickening out on this XDR-TB issue [1].
